# Stillbirth rates in low-middle income countries 2010 - 2013: a population-based, multi-country study from the Global Network

**DOI:** 10.1186/1742-4755-12-S2-S7

**Published:** 2015-06-08

**Authors:** Elizabeth M McClure, Sarah Saleem, Shivaprasad S Goudar, Janet L Moore, Ana Garces, Fabian Esamai, Archana Patel, Elwyn Chomba, Fernando Althabe, Omrana Pasha, Bhalachandra S Kodkany, Carl L Bose, Mabel Berreuta, Edward A Liechty, K Michael Hambidge, Nancy F Krebs, Richard J Derman, Patricia L Hibberd, Pierre Buekens, Albert Manasyan, Waldemar A Carlo, Dennis D Wallace, Marion Koso-Thomas, Robert L Goldenberg

**Affiliations:** 1 RTI International, Durham, NC, USA; 2 Aga Khan University, Karachi, Pakistan; 3 KLE University’s Jawaharlal Nehru Medical College, Belgaum, India; 4 Fundación para la Alimentación y Nutrición de Centro América y Panamá, Guatemala City, Guatemala; 5 Moi University School of Medicine, Eldoret, Kenya; 6 Lata Medical Research Foundation, Nagpur, India; 7 University Teaching Hospital, Lusaka, Zambia; 8 Institute for Clinical Effectiveness and Health Policy, University of Buenos Aires, Argentina; 9 University of North Carolina at Chapel Hill, Chapel Hill, NC, USA; 10 School of Medicine, University of Indiana, Indianapolis, IN, USA; 11 University of Denver School of Medicine, Denver, CO, USA; 12 Christiana Care Health Services, Newark, DE, USA; 13 Massachusetts General Hospital, Boston, MA, USA; 14 Tulane School of Public Health and Tropical Medicine, New Orleans, LA, USA; 15 University of Alabama at Birmingham, Birmingham, AL, USA; 16 Eunice Kennedy Shriver National Institute of Child Health and Human Development, Bethesda, MD, USA; 17 Department of Obstetrics and Gynecology, Columbia University School of Medicine, New York, NY, USA

**Keywords:** Stillbirth, low-middle income countries, obstetric care

## Abstract

**Background:**

Stillbirth rates remain nearly ten times higher in low-middle income countries (LMIC) than high income countries. In LMIC, where nearly 98% of stillbirths worldwide occur, few population-based studies have documented characteristics or care for mothers with stillbirths. Non-macerated stillbirths, those occurring around delivery, are generally considered preventable with appropriate obstetric care.

**Methods:**

We undertook a prospective, population-based observational study of all pregnant women in defined geographic areas across 7 sites in low-resource settings (Kenya, Zambia, India, Pakistan, Guatemala and Argentina). Staff collected demographic and health care characteristics with outcomes obtained at delivery.

**Results:**

From 2010 through 2013, 269,614 enrolled women had 272,089 births, including 7,865 stillbirths. The overall stillbirth rate was 28.9/1000 births, ranging from 13.6/1000 births in Argentina to 56.5/1000 births in Pakistan. Stillbirth rates were stable or declined in 6 of the 7 sites from 2010-2013, only increasing in Pakistan. Less educated, older and women with less access to antenatal care were at increased risk of stillbirth. Furthermore, women not delivered by a skilled attendant were more likely to have a stillbirth (RR 2.8, 95% CI 2.2, 3.5). Compared to live births, stillbirths were more likely to be preterm (RR 12.4, 95% CI 11.2, 13.6). Infants with major congenital anomalies were at increased risk of stillbirth (RR 9.1, 95% CI 7.3, 11.4), as were multiple gestations (RR 2.8, 95% CI 2.4, 3.2) and breech (RR 3.0, 95% CI 2.6, 3.5). Altogether, 67.4% of the stillbirths were non-macerated. 7.6% of women with stillbirths had cesarean sections, with obstructed labor the primary indication (36.9%).

**Conclusions:**

Stillbirth rates were high, but with reductions in most sites during the study period. Disadvantaged women, those with less antenatal care and those delivered without a skilled birth attendant were at increased risk of delivering a stillbirth. More than two-thirds of all stillbirths were non-macerated, suggesting potentially preventable stillbirth. Additionally, 8% of women with stillbirths were delivered by cesarean section. The relatively high rate of cesarean section among those with stillbirths suggested that this care was too late or not of quality to prevent the stillbirth; however, further research is needed to evaluate the quality of obstetric care, including cesarean section, on stillbirth in these low resource settings.

**Study registration:**

Clinicaltrials.gov (ID# NCT01073475)

## Background

Nearly 2.7 million third trimester stillbirths occur each year, most of which are thought to be preventable [1,2]. Globally there is a high variation in stillbirth rates with low-income sub-Saharan African and South East Asian countries reporting the highest rates, ranging from 20 – 40 per 1000 births, nearly 10 –fold higher than those documented in high-resource settings [3-5]. Nearly 98% of all stillbirths occur in low- and middle-income countries (LMIC), primarily in low-resource settings [1,6].

Within both high and low-resource settings, several common risk factors for stillbirths have been documented. Stillbirths are more likely to occur among those women who are of advanced age and, in some studies, teenage women are also at increased risk [5,7,8]. Women who are of lower socio-economic status, including those with less education, have been shown to be at increased risk [7,8]. Finally, women with prior pregnancy losses or with complicated pregnancies, including multiple gestations, have an increased risk of stillbirth [9].

Across all settings, poor quality health care is an important risk factor for stillbirth. Both the lack of access to antenatal care and lack of access to quality obstetric care have been associated with increased risk of stillbirth. In particular, mode of delivery may influence stillbirth risk [10,11]. While detailed cause of death data are mostly unavailable for stillbirth in low-income countries (LIC), in these settings recent efforts have been made to classify stillbirths by those with signs of maceration, suggesting a fetal death occurring in the antepartum period at least 12 hours prior to delivery, and those without signs of maceration, which are frequently intrapartum stillbirths or those which occur just prior to or during labor and delivery. This characterization is helpful in determining programmatic emphasis, as improved antenatal care may reduce antenatal stillbirths while improved obstetric labor and delivery care is needed to reduce those stillbirths occurring in the intrapartum period. In these settings with high stillbirth rates, studies have suggested that more than half of stillbirths may be intrapartum [12,13]. In contrast, in high-income countries (HIC), intrapartum stillbirths have largely been eliminated, suggesting that with adequate obstetric care, including cesarean section when indicated, most of these stillbirths are preventable [12].

While there has been increased attention to stillbirth in recent years, in LIC, there are still few population-based estimates of stillbirth rates, types of stillbirth, risk factors for stillbirth, or measures of health care associated with stillbirth. Demographic health surveys generally have excluded stillbirth as routine pregnancy outcomes, while research studies have primarily been conducted in hospital-based settings. Thus, we sought to determine population-based stillbirth rates in low-resource settings in 6 countries participating in the *Eunice Kennedy Shriver* National Institute of Child Health and Human Development Global Network for Women’s and Children’s Health Research (Global Network) [14]. We also evaluated the maternal characteristics and obstetric and antenatal care associated with risk of stillbirth, with exploration of factors associated with macerated and intrapartum stillbirths.

## Methods

The Global Network’s Maternal Newborn Health Registry (MNHR) is a prospective, population-based observational study which includes all pregnant women and their outcomes in defined geographic communities (clusters). These clusters were established with approximately 300 – 500 annual births in sites in western Kenya, Zambia (Kafue and Chongwe), Pakistan (Thatta), India (Belgaum and Nagpur), Guatemala (Chimaltenango), and Argentina (Corrientes). The MNHR was initiated at each of the study sites between 2008 and 2009 and continues to the present, except in Argentina, which ended data collection in March 2013.

Registry administrators (RAs), paid study staff who were usually community health workers or nurses, identified pregnant women and generally consented those who were eligible by 20 weeks gestation. All women who were residents of the defined communities were eligible and contacted. The RAs obtained basic health information at enrollment, and then conducted a follow-up visit following delivery to collect pregnancy outcomes and health care provided during delivery. Information on the study outcomes was based on medical record review, and birth attendant and family interviews.

Stillbirth was defined using a modified World Health Organization (WHO) criteria of fetal deaths occurring at ≥20 weeks gestation (or for those without gestational age available ≥500 g) [15]. The stillbirth rate was calculated as the number of stillbirths per 1,000 births, the sum of live births and stillbirths. Macerated stillbirths were defined as those with signs of maceration at delivery including skin and soft-tissue changes such as skin discoloration, redness, sloughing of skin, and overriding of cranial sutures. (The RAs were trained to recognize stillbirths with maceration using both descriptions and pictures of fetuses with this condition.) Those without maceration were considered to be intrapartum stillbirths; however, while we assumed that those stillbirths with maceration had died prior to labor and did not have fetal heart tones, in this data set, we did not confirm that those stillbirths without maceration had fetal heart tones in labor. For maternal characteristics among those with multiple births, women’s status regarding a ‘stillbirth’ or ‘live birth’ was determined by the status of her firstborn of the multiple gestation.

## Data analyses

All data were entered and edited locally at a research computer at each study site. Data were transmitted using a secure process, with additional edits performed at the data coordinating center, RTI International (RTI). Descriptive analyses included calculating the frequency and distribution of values. Additional analyses were performed to determine the relative risk of having a stillbirth with factors previously associated with stillbirth based on literature review, using general estimation equations to adjust for the study clusters (study site). All data were analyzed using SAS v.9.3 (Cary, NC).

## Ethics approval

This study was reviewed and approved by all sites’ ethics review committees (Instituto de Efectividad Clínica y Sanitaria, University of Buenos Aires, Argentina; Francisco Marroquin University, Guatemala; University of Zambia, Biomedical Research Ethics Committee, Zambia; Moi University, Kenya; Aga Khan University; KLE University’s Jawharal Nehru Medical College, Belgaum; Indira Gandhi Medical College, Nagpur), the institutional review boards at each U.S. partner university and the data coordinating center (RTI International). All women provided informed consent for data collection and follow-up visits.

## Results

**Figure 1:**
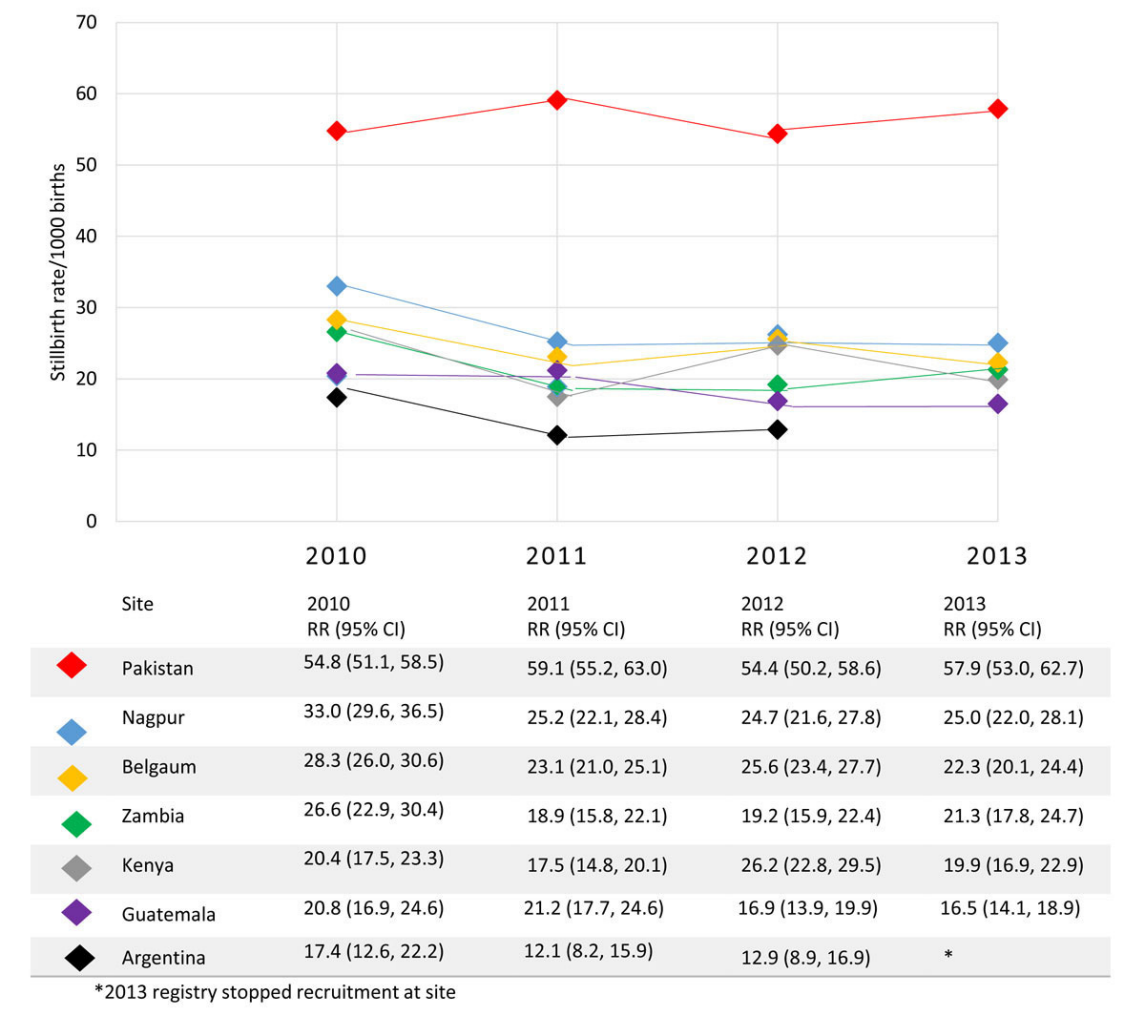
Stillbirth rates by Global Network site, 2010-2013

During the study period from January 2010 through December 2013, a total of 269,614 women were enrolled. These women had 272,089 births, of which 7,865 were stillbirths. The overall stillbirth rate for the entire study period was 28.9 per 1000 births. The stillbirth rates ranged from 13.6 in Argentina to 56.5 per 1,000 births in Pakistan. The India sites had significant reductions in stillbirth rates from 2010 to 2013 (p<0.05), while the rates in Latin American and African sites decreased and the Pakistan site rates increased, though neither reached statistical significance. Figure 1 illustrates the stillbirth rates over time by study site.

Table 1 describes the maternal characteristics and risks for stillbirth. Women who were >35 years of age were more likely to have a stillbirth (RR 1.6, 95% CI 1.5, 1.8) compared with women 20-35 years of age. Women without formal education were also more likely to deliver stillbirths (RR 1.9, 95% CI 1.7, 2.2), compared to those with higher education. Finally, those with parity >2 were more than twice as likely to have a stillbirth as those with those with parity 1-2 (RR 1.4, 95% CI 1.3, 1.5). Those who had experienced a prior pregnancy loss were also at higher risk for delivering a stillbirth compared with those without prior loss (RR 2.4, 95% CI 2.2, 2.7).

**Table 1:** Maternal characteristics associated with stillbirth

	Stillbirth	Live birth	RR for SB vs. LB (95% CI)
Mother’s enrolled, N	7,624	261,990	

Maternal age			

<20	732 (9.6)	31,729 (12.1)	1.0 (1.0, 1.1)

20-35	6,423 (84.4)	219,821 (84.0)	1.0

>35	454 (6.0)	10,020 (3.0)	1.6 (1.5, 1.8)

			

Education			

No formal education	3,151 (41.5)	64,284 (24.6)	1.9 (1.7, 2.2)

Primary	2,281 (30.0)	99,567 (38.2)	1.3 (1.2, 1.5)

Secondary	1,776 (23.4)	78,329 (30.0)	1.2 (1.1, 1.3)

University+	383 (5.0)	18,619 (7.1)	1.0

			

Parity			

0	2609 (34.3)	87,908 (33.7)	1.3 (1.2, 1.3)

1-2	2623 (34.5)	110,597 (42.3)	1.0

>2	2370 (31.2)	62,783 (24.0)	1.4 (1.3, 1.5)

			

Prior loss			

No	4,277 (85.7)	163,142 (94.2)	1.0

Yes	712 (14.3)	10,064 (5.8)	2.4 (2.2, 2.7)

We also evaluated the antenatal and delivery care in relationship to stillbirth (Table 2). Women with no prenatal care had an increased risk of stillbirth (RR 3.0, 95% CI 2.5, 3.7). Those who had not received testing for syphilis (RR 1.6 95% CI 1.5, 1.8), HIV (RR 2.5, 95% CI 2.1, 2.9) or who had not received tetanus toxoid (RR 2.2 95% CI 2.0, 2.5) were at increased risk of stillbirth relative to those women who had received the testing. Women delivered by a family member or other non-healthcare provider were at increased risk of stillbirth (RR 2.8, 95% 2.2, 3.5). Higher level of care was not associated with reduced risk of stillbirth. Finally, those women delivered by cesarean section had decreased risk of stillbirth (RR 0.6, 95% CI 0.6, 0.7) compared to those with a spontaneous vaginal delivery, while women with a vaginal assisted delivery were at increased risk of stillbirth (RR 2.0, 95% CI 1.3, 3.1) compared to those with a vaginal delivery.

**Table 2:** Antenatal and delivery care characteristics and risk of stillbirth, 2010-2013

	Stillbirth, N (%)	Live birth, N (%)	Relative Risk (95% CI)*
ANC Visits			

0	331 (8.2)	3,659 (2.6)	3.0 (2.5, 3.7)

1-2	1,358 (33.5)	25,842 (18.3)	1.0

3+	2,370 (58.4)	111,554 (79.1)	2.0 (1.7, 2.4)

Syphilis testing received			

Yes	1,917 (25.4)	103,889 (39.9)	1.0

No	5,619 (74.6)	156,282 (60.1)	1.6 (1.5, 1.8)

HIV testing received			

Yes	4,199 (55.6)	195,255 (74.8)	1.0

No	3,359 (44.4)	65,666 (25.2)	2.5 (2.1, 2.9)

Tetanus toxoid vaccine			

Yes	5,352 (70.4)	225,165 (86.1)	1.0

No	2,250 (29.6)	36,396 (13.9)	2.2 (2.0, 2.5)

Birth attendant, N (%)			

Physician	3,155 (41.4)	100,662 (38.4)	1.0

Nurse/Midwife/HW	1,851 (24.3)	86,444 (33.0)	0.6 (0.6, 0.7)

TBA	1,404 (18.4)	62,573 (23.9)	0.5 (0.4, 0.6)

Family/Other	1,202 (15.8)	12,300 (4.7)	2.8 (2.2, 3.5)

Delivery location			

Hospital	3,598 (47.2)	120,433 (46.0)	1.0

Clinic	1,415 (18.6)	66,929 (25.6)	0.6 (0.5, 0.7)

Home/Other	2,609 (34.2)	74,554 (28.5)	1.0 (0.8, 1.2)

Delivery mode			

Vaginal	6,238 (88.4)	225,418 (86.0)	1.0

Vaginal assisted	282 (4.0)	3,899 (1.5)	2.0 (1.3, 3.1)

C-section	533 (7.6)	32,665 (12.5)	0.6 (0.6, 0.7)

*Relative risk accounted for study cluster (site)

**Figure 2:**
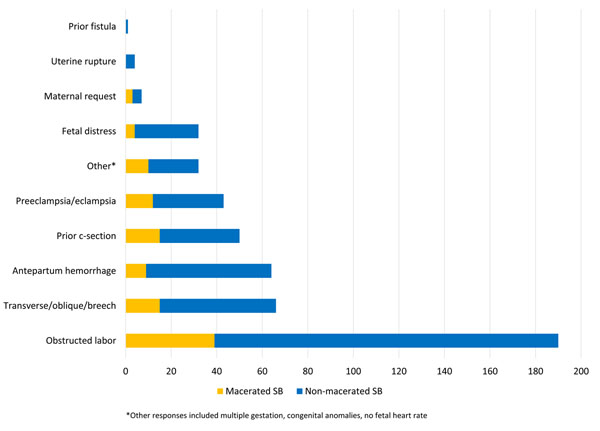
Indications for cesarean section among fresh and macerated stillbirth for Global Network sites, 2010 - 2013

Because cesarean sections are generally not indicated for women with a known stillbirth, to further assess the quality of obstetric care, we explored the indications for cesarean section among women who delivered a stillbirth. A total of 533 (7.6%) women with stillbirths were delivered by cesarean section. Figure 2 illustrates the indication for cesarean section for those who ultimately delivered a stillbirth. The most common indication for cesarean section among those with stillbirth was obstructed labor (n=190), followed by malposition including breech (n=66) and major hemorrhage (n=63). Of those delivered by cesarean section, 132 were macerated (5.6% of all of the macerated stillbirths) and 438 were non-macerated (90% of non-macerated).

Compared to live births, the stillbirths were more likely to be preterm (RR 12.4, 95% CI 11.2, 13.6) and were also more likely to be low birth weight (RR 12.1 95% CI 10.8, 13.5) (Table 3). They were also more likely to be male (RR 1.2, 95% CI 1.1, 1.2) and from a multiple gestation (RR 2.8, 95% CI 2.4, 3.2). Those with major congenital anomalies (RR 9.1, 95% CI 7.3, 11.4) had a higher risk of stillbirth. Women with a breech presentation or transverse lie also had a higher risk of stillbirth (RR 3.0, 95% 2.6, 3.5).

**Table 3:** Fetal characteristics and risk for stillbirth, 2010-2013

	Stillbirth, N (%)	Live Birth, N (%)	Relative risk for stillbirth vs. livebirth (95% CI) *
Estimated gestational age			

Preterm (< 37 weeks)	4,147 (59.4)	22,912 (8.9)	12.4 (11.2, 13.6)

Term (≥37 weeks)	2,830 (40.6)	233,556 (91.1)	1.0

Birth weight			

< 2500g	4,654 (64.4)	29,993 (11.4)	12.1 (10.8, 13.5)

≥ 2500g	2,570 (35.6)	234,056 (88.6)	1.0

Gender			

Male	3,856 (55.6)	136,886 (51.8)	1.2 (1.1, 1.2)

Female	3,083 (44.4)	127,275 (48.2)	1.0

Multiple gestation			

Yes	383 (5.0)	4,457 (1.7)	2.8 (2.4, 3.2)

No	7,254 (95.0)	259,674 (98.3)	1.0

Congenital anomaly			

Yes	330 (4.7)	1,091 (0.4)	9.1 (7.3, 11.4)

No	6,672 (95.3)	259,098 (99.6)	1.0

Breech presentation			

Yes	553 (7.1)	5,749 (2.2)	3.0 (2.6, 3.5)

No	7,255 (92.9)	257,933 (97.8)	1.0

*Relative risks accounted for study cluster (site)

**Figure 3:**
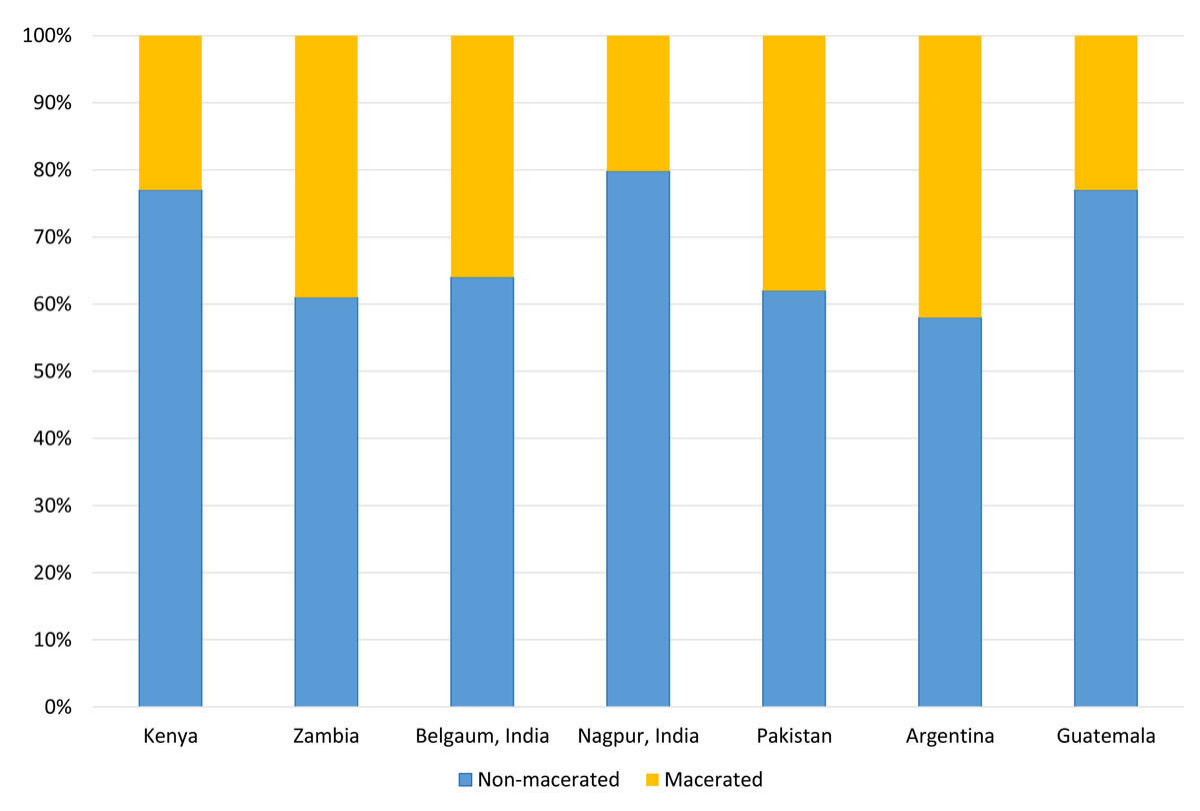
Proportion of macerated and non-macerated stillbirth by Global Network site, 2010-2013

Finally, we assessed the percentage of non-macerated and macerated stillbirths by site (Figure 3). A total of 4,872 (67%) of the stillbirths were non-macerated. The percentage of non-macerated stillbirth ranged from 78% in Nagpur, India to 58% in Argentina.

## Discussion

In this cohort study conducted in low-resource settings across six countries in Asia, sub-Saharan Africa and Latin America, the overall stillbirth rate was 28.9 per 1000 births. Since 2010, the stillbirth rates decreased in the Latin American and Indian sites, with modest decreases in the Zambia or Kenya sites and increasing stillbirth rates in the Pakistan site over the four-year period. The overall stillbirth rates remained relatively high, ranging from 13.6 in Argentina to 56.5 per 1,000 births in Pakistan. These rates are consistent with other modeling estimates [1].

This is one of the largest prospective studies of stillbirth in low-resource settings in low to upper-middle income countries conducted to date. One of the study strengths was that we obtained population-based outcomes with excellent coverage of the catchment areas of the study communities, including very high enrollment and 98% follow-up to obtain pregnancy outcomes. Furthermore, the study reflects four years of data. One of the study’s limitations was that while we obtained signs of maceration, these were through observation or report, which previous studies have suggested may over-represent intrapartum stillbirth [16]. We were also unable to reliably capture the cause of stillbirth, given the high proportion of births delivered outside of health care settings. However, the study did collect basic demographic data and health care utilization by women, cared for in a range of health care settings, with one-third delivered at home. As described elsewhere, related research was conducted over the study period in which training and supervision occurred to help ensure detection of stillbirth and differentiation of macerated from non-macerated stillbirths [17-19].

The maternal socio-economic and maternal risk factors for stillbirth observed were similar to those documented in low-middle income country studies conducted elsewhere [3-5,7-10]. That is, women who had less education and who were older were at significantly higher risk for stillbirth. Similarly, those women having less access to antenatal care, as documented through fewer ANC visits and reduced rates of prenatal testing, had increased risk for stillbirth. While the rates of HIV and syphilis testing were associated with the stillbirth rate, we assume the relationship is primarily explained by these tests indicating the adequacy of antenatal and obstetric care. Similarly, provision of tetanus immunization, which has not been associated with stillbirth, is also a marker for the adequacy of care.

Being preterm and of low birth weight were both associated with more than ten-fold risks of being a stillbirth compared to a live birth. In this case, it was not possible to distinguish whether the preterm labor increased risk for stillbirth or whether the condition that caused the stillbirth may have also precipitated the early delivery. Regardless, prematurity and low birth weight were strongly associated with stillbirth.

Women with complicated pregnancies, including those with multiple gestations and breech presentations, were at significantly increased risk for stillbirth in our study. Similarly, those with major congenital anomalies had increased of stillbirth, similar to risks shown from other LMIC [4]. Across the Global Network sites, non-macerated stillbirths represented about 67% of all stillbirths. While this rate is higher than some other LMIC studies, it is consistent with studies suggesting that intrapartum stillbirth rates are highest in the geographic regions with high stillbirth rates [13]. This is an important relationship, as an intrapartum stillbirth suggests that the fetus was alive at time of delivery care, that the stillbirth likely occurred in association with intrapartum asphyxia or trauma, and that the stillbirth may have been preventable [12,20]. With the high proportion of intrapartum stillbirths, our results suggest that stillbirth rates could be substantially reduced by focusing on these deaths with improved obstetric labor and delivery care. While many could be prevented with improved care, a proportion of non-macerated stillbirths are associated with other causes such as major congenital anomalies [21]. Our results also showed overall higher stillbirth rates at hospitals and with physicians, relative to the stillbirth rates found at lower levels of care. Because this study was conducted at the population level, this finding is likely to be associated with the higher risk deliveries presenting at these higher levels of care [21]; however, it may also have implications for the quality of care available at some of those facilities [12,22].

Prior research has suggested that timely access to high-quality care at delivery can substantially reduce stillbirth, especially those occurring in the intrapartum period, including those associated with many types of complicated pregnancies [20,23,24]. We explored this issue through examining cesarean section and stillbirth further and found that altogether 8% of all women with a stillbirth were delivered by cesarean section. Because cesarean section is generally not indicated where there is a fetal death [25-27], we examined the indications and noted that the majority of these cesarean sections were conducted for obstructed labor, major antepartum hemorrhage, or malposition. While these conditions are considered appropriate cesarean section indications [25], in these settings the cesarean section was inadequate or performed too late to save the fetus. The fact that such a high proportion of women with stillbirths had cesarean section suggests that the primary motivation may have been to save the mother’s life. However, further research would be needed to confirm this speculation. Additionally, while research in HIC has suggested that stillbirths associated with these types of complications are generally preventable with quality obstetric care, this finding would also need to be further explored in low-resource areas.

Tools such as perinatal audits have been shown to improve quality of facility care and reduce stillbirth [28]. Quality of care includes the judgment to determine which women are at risk and require interventions such as cesarean section, and performing these interventions well. However, in addition to the quality of obstetric care, the timeliness of providing obstetric care is critical, especially to save the fetus. As suggested by our results, cesarean section performed late may not benefit the fetus or poorly performed instrumental delivery may increase risk. Furthermore, the increased mortality associated with hospital and physician deliveries suggested that women with risk may have been seeking care; however, the care may have been delivered too late to save the fetus.

## Conclusions

In summary, this analysis presents stillbirth rates in LMIC which are substantially higher than in HIC, with a wide range of increased rates. The majority of stillbirths were not macerated, suggesting that they occurred at or near the time of delivery. Many of these stillbirths were likely to be preventable by better obstetric care. Strategies to improve antenatal and obstetric labor and delivery care for women in order to substantially reduce stillbirths, while well documented to reduce stillbirth in HIC, may not be easily transferrable and have yet to be proven effective in low-resource settings. Given the high rates of stillbirth and slow rates of reduction found across the diverse settings in LMIC, we believe further research is needed to evaluate the strategies necessary to substantially reduce stillbirth in low-resource countries.

## List of abbreviations used

ANC: Antenatal care; FSB: Fresh stillbirth; HIC: high-income countries; LIC: Low-income countries; LMIC: Low-middle income countries; MNHR: Maternal and newborn health registry; MSB: Macerated stillbirth; WHO: World Health Organization.

## Competing interests

The authors declare that they have no competing interests.

## Authors’ contributions

EMM and RLG conceived of the study and wrote the first draft. EMM, RLG, SS, SSG, FE, AG, AP, EC, JB, OP, BK, CLB, EAL, KMH, NFK, RJD, PLH, PB, WAC and MKT developed the study protocol. FA, SSG, AG, FE, AP, AM oversaw field activities and performed data monitoring. CLB, SSG, WAC, SSG, FE, and KMH provided edits to the manuscript. JLM, DDW, and EMM performed data analyses. All authors read and approved the final manuscript.

## Peer review

Reviewer reports for this article can be found in Additional file 1.

## Supplementary Material

Additional file 1 Click here for file

## Declarations

This article has been published as part of *Reproductive Health* Volume 12 Supplement 2, 2015: Research reports from the NICHD Global Network for Women’s and Children’s Health Research Maternal and Newborn Health Registry. The full contents of the supplement are available online at http://www.reproductive-health-journal.com/supplements/12/S2. Publication of this supplement was supported by grants from the Eunice Kennedy Shriver National Institute of Child Health and Human Development to RTI International.
