# Supplementary material for: Stillbirth rates in low-middle income countries 2010 - 2013: a population-based, multi-country study from the Global Network
Source: Reprod Health. 2015 Jun 8;12(Suppl 2):S7. doi: 10.1186/1742-4755-12-S2-S7 (PMC4464024; doi:10.1186/1742-4755-12-S2-S7)
Supplement: Additional file 1 [file 1742-4755-12-S2-S7-S1.pdf]

Referee's comments to the authors– this sheet WILL be seen by the author(s) and published with the article

Title	Stillbirth trends in low-middle income countries 2010 - 2013: A population-based, multi-country study from the Global Network
Author(s)	Elizabeth M McClure, Sarah Saleem, Shivaprasad S Goudar, Janet L Moore, Ana Garces, Fabian Esamai, Archana Patel, Elwyn Chomba, Omrana Pasha, Bhalachandra S Kodkany, Carl L Bose, Edward A Liechty, K Michael Hambidge, Nancy F Krebs, Richard J Derman, Patricia L Hibberd, Pierre Buekens, Albert Mayasyan, Waldemar A Carlo, Dennis D Wallace, Marion Koso-Thomas and Robert L Goldenberg
Referee's name	Jan Jaap Erwich

When assessing the work, please consider the following points, where applicable:

1. Is the question posed by the authors new and well defined?
2. Are the methods appropriate and well described, and are sufficient details provided to replicate the work?
3. Are the data sound and well controlled?
4. Does the manuscript adhere to the relevant standards for reporting and data deposition?
5. Are the discussion and conclusions well balanced and adequately supported by the data?
6. Do the title and abstract accurately convey what has been found?
7. Is the writing acceptable?

Please make your report as constructive and detailed as possible in your comments so that authors have the opportunity to overcome any serious deficiencies that you find and please also divide your comments into the following categories:

- Major Compulsory Revisions (which the author must respond to before a decision on publication can be reached)
- Minor Essential Revisions (such as missing labels on figures, or the wrong use of a term, which the author can be trusted to correct)
- Discretionary Revisions (which are recommendations for improvement but which the author can choose to ignore)

Where possible please supply references to substantiate your comments.

When referring to the manuscript please provide specific page and paragraph citations where appropriate.

General comments: This is a well written manuscript on an important topic. Unique population based stillbirth rates have been collected. The team is well known in the field, and have done a good job. Limitations are well described. I have only a few minor suggestions.

Major compulsory revisions: none

Minor essential revisions: none

Discretionary revisions:

1. Title: "trends" could be interpreted differently, change into or add rates, or numbers? Changes in Stillbirth numbers in low…..
2. Abstract: ln2: 98% of stillbirths worldwide…
3. Non-macerated, has not such a direct relation with cause, as the authors mention themselves, e.g. they can have lethal abnormalities, or placental abruption. Not all are preventable only by improving care (unfortunately). Some more explanation could be usefull.
4. Major congenital malformation give RR of 9.1. How were they diagnosed? Usually this is not so much a cause for stillbirth as well for neonatal death.
5. Which definition for stillbirth was used, gestational weeks? Birth weight?
6. Pg 5 ln 9: A/ macerated….. what does the A/means?
7. In the text (bottom pg 5 and top pg 6) there are the results described identical as in the tables. This doubles, if space is not an issue, no problem, otherwise choose for text or table (I prefer the tables).

Referee's comments to the authors– this sheet WILL be seen by the author(s) and published with the article

Title	Stillbirth trends in low-middle income countries 2010 - 2013: A population-based, multi-country study from the Global Network
Author(s)	Elizabeth M McClure, Sarah Saleem, Shivaprasad S Goudar, Janet L Moore, Ana Garces, Fabian Esamai, Archana Patel, Elwyn Chomba, Omrana Pasha, Bhalachandra S Kodkany, Carl L Bose, Edward A Liechty, K Michael Hambidge, Nancy F Krebs, Richard J Derman, Patricia L Hibberd, Pierre Buekens, Albert Mayasyan, Waldemar A Carlo, Dennis D Wallace, Marion Koso-Thomas and Robert L Goldenberg
Referee's name	Michael Varner

When assessing the work, please consider the following points, where applicable:

1. Is the question posed by the authors new and well defined?
2. Are the methods appropriate and well described, and are sufficient details provided to replicate the work?
3. Are the data sound and well controlled?
4. Does the manuscript adhere to the relevant standards for reporting and data deposition?
5. Are the discussion and conclusions well balanced and adequately supported by the data?
6. Do the title and abstract accurately convey what has been found?
7. Is the writing acceptable?

Please make your report as constructive and detailed as possible in your comments so that authors have the opportunity to overcome any serious deficiencies that you find and please also divide your comments into the following categories:

- Major Compulsory Revisions (which the author must respond to before a decision on publication can be reached)
- Minor Essential Revisions (such as missing labels on figures, or the wrong use of a term, which the author can be trusted to correct)
- Discretionary Revisions (which are recommendations for improvement but which the author can choose to ignore)

Where possible please supply references to substantiate your comments.

When referring to the manuscript please provide specific page and paragraph citations where appropriate.

General comments:
This is a descriptive report of stillbirths occurring in the context of a population-based birth cohort from the NICHD Global Network. The number of cases is impressive and confirms a number of associations with stillbirth that have been previously reported. Stillbirth rates are higher in women with little or no education, who are older and who have little or no access to antenatal or intrapartum care. In this large series stillbirths were more likely to be preterm, complicated by major anomalies and malpresentation, or part of a multi-fetal gestation. The authors utilize the presence or absence of maceration as a proxy for antenatal versus intrapartum etiologies. Although antenatal and intrapartum stillbirth do have some etiologies in common, the development of intervention strategies would be different.

It would be helpful to have more details about how maceration was defined and how accurate this diagnosis really was. It would also be helpful if the authors could at least speculate on how these findings might drive subsequent health policies.

(continue on the next sheet)

Continued:

Major compulsory revisions:
1. A specific definition of macerated stillbirth should be provided. To be told that a 'non-macerated stillbirth is one without evidence of maceration' (third paragraph of Methods) is not satisfactory. In the second paragraph of the Discussion we are told that signs of maceration were made 'through observation or report' and that 'training and supervision occurred to help ensure detection of stillbirth and differentiation of macerated and non-macerated (refs 17-19)'. In the current version of this manuscript these represent new data that should be included in the Methods.

2. The last sentence of the third paragraph of the Methods section ('For maternal characteristics, . . .') is unclear, at least to this reviewer. Does this refer to the first-born fetus of a multiple gestation? To a woman's first birth in the authors' registry? Just to primigravidas?

3. In the first paragraph of the Results section we are told that the stillbirth rates in Pakistan increased over the study period but that the rates decreased in the other sites. Were any of these changes statistically significant? Without such confirmation one can really only describe an apparent trend. This concern arises again in the final sentence of the manuscript in which the authors state thee was a 'lack of reductions in stillbirth rates found across the diverse settings in LMIC'. In this case, does this refer to the current data or is it a more global observation?

4. Did the prevalence of macerated versus non-macerated stillbirth vary with gestational age? This information could be helpful for developing more focused preventive strategies.

Minor essential revisions:
1. The authors alternately define their study populations as low-middle income and low-income countries. This should be consistent.

2. Why are no 2013 data available for Argentina in 2013 (Figure 1)?

3. In the third paragraph of the Results several associations are described between syphilis and HIV testing and tetanus immunization and stillbirth. We are never told, either in the Results or the Discussion, whether the authors believe this is a cause-and-effect relationship or a marker for some other underlying etiology.

Discretionary revisions:
1. The final sentence of the Conclusion of the Abstract begins with 'The relatively high rate of cesarean section suggested . . .' It is not clear whether this is the overall cesarean rate for the combined populations or just for stillbirths. While the overall cesarean rate is eventually provided in Table 2, individuals who only read the Abstract might find this confusing.
